# Mid- and Late-Life Migraine Is Associated with an Increased Risk of All-Cause Dementia and Alzheimer’s Disease, but Not Vascular Dementia: A Nationwide Retrospective Cohort Study

**DOI:** 10.3390/jpm11100990

**Published:** 2021-09-30

**Authors:** Hyun-Joo Lee, Hyunjae Yu, Son Gil Myeong, Kijoon Park, Dong-Kyu Kim

**Affiliations:** 1 Division of Big Data and Artificial Intelligence, Institute of New Frontier Research, Chuncheon Sacred Heart Hospital, Hallym University College of Medicine, Chuncheon 24252, Korea; leekul79@gmail.com (H.-J.L.); yunow@hallym.or.kr (H.Y.); 2 Department of Otorhinolaryngology—Head and Neck Surgery, Chuncheon Sacred Heart Hospital, Hallym University College of Medicine, Chuncheon 24252, Korea; ssadoong2@hallym.or.kr (S.G.M.); voix712@hallym.or.kr (K.P.)

**Keywords:** migraine disorders, dementia, Alzheimer disease, headache, cohort studies

## Abstract

We used a nationwide cohort sample of data from 2002 to 2013, representing approximately 1 million patients to investigate the prospective association between migraine and dementia. The migraine group (*n* = 1472) included patients diagnosed between 2002 and 2004, aged over 55 years; the comparison group was selected using propensity score matching (*n* = 5888). Cox proportional hazards regression analyses was used to calculate the hazard ratios (HRs). The incidence of dementia was 13.5 per 1000 person-years in the migraine group. Following adjustment for sociodemographic and comorbidities variables, patients with migraine developed dementia more frequently than those in the comparison group (adjusted HR = 1.37, 95% confidence interval [CI], 1.16–1.61). In the subgroup analysis, we found a higher HR of dementia events in male, the presence of comorbidities, and older age (≥65) patients with migraine, compared to those without migraine. Moreover, patients with migraine had a significantly higher incidence of Alzheimer’s disease (adjusted HR = 1.31, 95% CI, 1.08–1.58), but not vascular dementia, than those without migraine. Therefore, our findings suggest that mid- and late-life migraines may be associated with an increased incidence of all-cause dementia and Alzheimer’s disease, but not vascular dementia.

## 1. Introduction

A migraine is a headache that can cause severe throbbing pain or a pulsing sensation, usually on one side of the head and may be aggravated by physical activity [1]. Severe cases can affect a person’s daily life, including their ability to work or study. Dementia usually occurs in later life and is characterized by slow progressive memory loss, impaired cognitive function, and the inability to perform activities of daily living [2]. These neurological diseases (migraine and dementia) place a considerable burden on public health. Additionally, some studies have indicated that the vascular change in migraine could contribute to dementia development since cerebral small vessel disease could increase the risk for cerebrovascular dysfunction [3,4]. However, the literature regarding the relationship between migraine and dementia are controversial [5,6,7,8,9,10].

One prior meta-analysis demonstrated that any type of headache showed a significantly increased risk of all-cause dementia, but there is no association between any type of headache and incident Alzheimer’s disease [5]. In contrast to this study, other recent studies revealed a positive association of migraine with all-cause dementia as well as Alzheimer’s disease [6,7,8]. However, these studies had several limitations, including those based on small populations (not nationwide population), only involving older individuals (not including those of middle age), and without a clear temporal separation between migraine and dementia. Moreover, another recent cohort study from the USA demonstrated that this study did not show the association of migraine with incident dementia [9]. Thus, to date, the association between migraine and dementia has still unclear.

Therefore, to determine a possible link between migraine and all types of dementia, we analyzed the prospective development of dementia events in patients with migraines using a nationally representative sample from the National Sample Cohort (NSC) data obtained from the Korean National Health Insurance Service (KNHIS) in South Korea.

## 2. Materials and Methods

### 2.1. Study Setting and Participants

**Figure 1:**
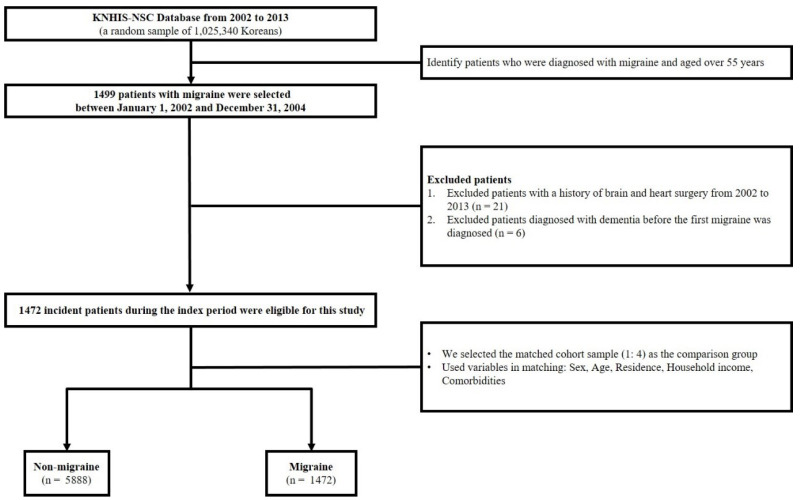
Schematic description of study design.

Among 1,025,340 patient datasets, the number of patients with migraine was identified according to the International Classification of Diseases, 10th revision (ICD-10), code. The migraine group included all patients who received an initial diagnosis of migraine (G43) during January 2002 and December 2004 at an age over 55 years at cohort entry. To further improve the accuracy of the migraine definition, we exclusively included patients who had been diagnosed with migraine more than three times between 2002 and 2004 and were diagnosed by neurologists. Patients were excluded if they (1) were diagnosed as dementia between 2002 and 2004, (2) died as a result of any cause between 2002, and (3) had a history of brain or heart surgery between 2002 to 2013. We randomly selected propensity score-matched (4:1) participants to establish the comparison group (non-migraine) from the remaining cohort registered in the database. In this study, we have matched comparison subjects to patients with migraines based on sociodemographic factors (age, sex, residential area, household income), comorbidities, and the enrollment year (migraine diagnosis). Figure 1 showed the schematic flow of the study design.

### 2.2. Study Outcome

The health claims data of all participants were examined for the development of dementia (Alzheimer’s disease [F00, G30], vascular dementia [F01], and others [F02, F03]) until December 2013. We included only dementia patients who were diagnosed by neurologists. In this study, the endpoints were an event (dementia) or all-cause mortality. However, if patients had no events and were alive on December 31, 2013 (the final date of data collection), we censored this time point (Supplementary Table S1). The risk of dementia in migraine patients and the comparison groups was compared as person-years at risk. The duration was defined as between either the date of dementia diagnosis (the migraine group) or the enrollment year (the comparison group) and the patient’s respective endpoint.

### 2.3. Independent Variables

Table 1 presents the patient characteristics, including sex, age, residence, household income, disability, and comorbidities. This study was categorized into three age groups (55–64, 65–74, and ≥75 years), three household income groups (low: ≤30%, middle: 30.1–69.9%, and high: ≥70% of the median), and three residential areas (1st area: Seoul, the largest metropolitan region; 2nd area: other metropolitan cities; and 3rd area: remaining areas). Data on comorbidities, including hypertension (I10–I15), diabetes mellitus (E10–E14), stroke (I60–I63), chronic kidney disease (N18), and disorders of lipoprotein metabolism and other lipidemias (E78) were obtained using on the relevant diagnostic code. We defined the presence of comorbidities as any diagnoses of these codes between 2002 and 2004 prior to the diagnosis of migraine.

### 2.4. Statistical Analyses

We constructed the cohort sample by one-to-four propensity score matching and determined the incidence rate per 1000 person-years for dementia. To identify whether migraine increased the risk of dementia, we calculated the hazard ratio (HR) and 95% confidence interval (CI) using the Cox proportional hazard regression. Additionally, subgroup analysis was performed according to sex, age, the presence of comorbidities. The overall specific disease-free time for the entire observation period was described using Kaplan–Meier survival curves. We used the R software program (R Foundation for Statistical Computing, Vienna, Austria) for all statistical analyses, with a significance level of 0.05.

## 3. Results

### 3.1. Effect of Migraine on the Incidence of Dementia in Patients Aged over 55 Years

**Figure 2:**
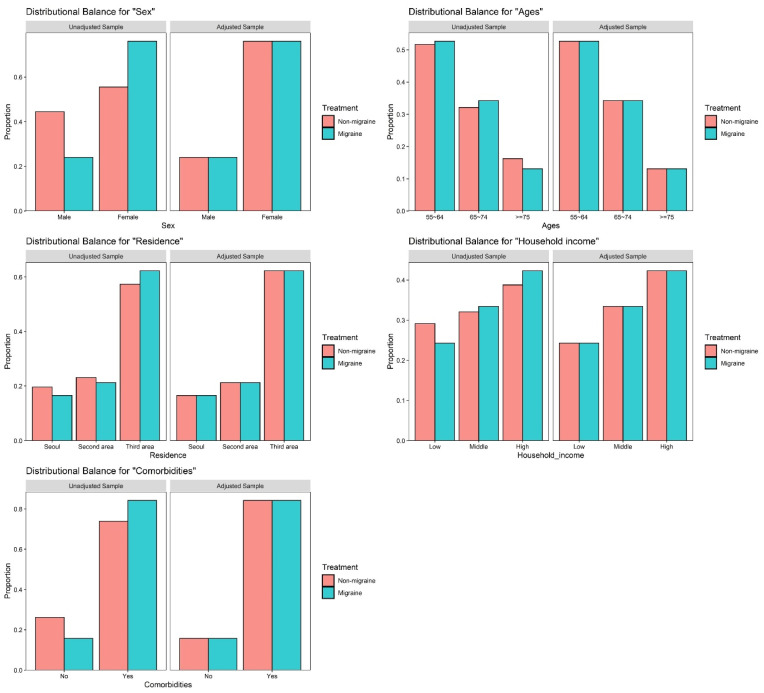
Balance plot for 5 variables before and after matching.

The present study comprised 1472 patients who were diagnosed with migraine and 5888 matched participants who were not diagnosed with migraine. Table 1 showed the characteristics of the study population, for the migraine and comparison group. We found similar distributions between the two groups, given that each variable was appropriately matched (Figure 2). The overall incidence of dementia was higher in patients who were diagnosed with migraine (13.5 per 1000 person-years) than in the comparison group (11.1 per 1000 person-years) (Table 2).

### 3.2. Hazard Ratios of Dementia in Patients with Migraine Aged over 55 Years

On the analysis of the Cox regression model, we found that migraine patients aged over 55 years was linked with prospective dementia development as 1.37 adjusted HR (95% CI, 1.16–1.61) during the follow-up period (Table 2).

In the subgroup analysis, we found higher adjusted HR of the prospective development of dementia in male patients with migraine (adjusted HR = 2.07, 95% CI 1.44–2.99), compared to the female those (Table 3). We also observed a significantly higher likelihood of developing dementia in migraine patients with a history of comorbidities (adjusted HR = 1.38, 95% CI 1.17–1.64) than those without a history of comorbidities (Table 4), and that the adjusted HR for developing dementia in patients with migraine was higher in old age (Table 5). Moreover, we detected that the adjusted HR of developing Alzheimer’s disease in patients aged over 55 years who have been diagnosed with dementia was 1.31 (95% CI 1.08–1.58), compared to the comparison group; however, we could not find any association with vascular dementia (adjusted HR = 1.42, 95% CI 0.98–2.05) in patients aged over 55 years who were diagnosed with dementia (Table 6).

**Figure 3:**
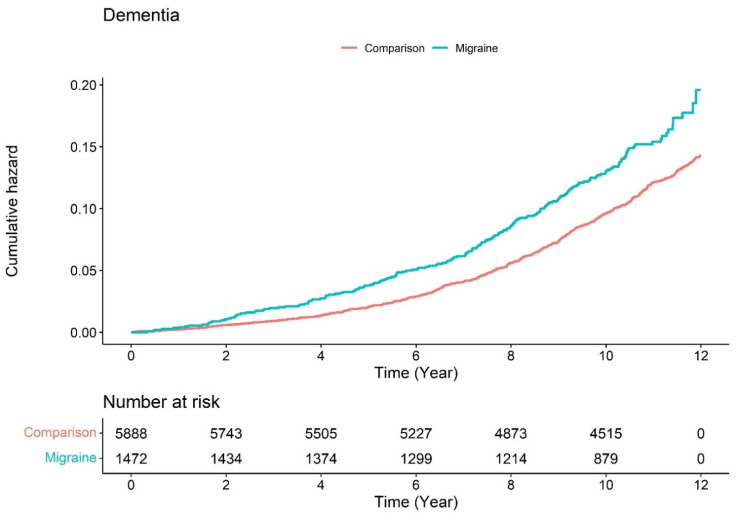
Cumulative hazard plot of specific between migraine and comparison (participants without migraine).

Figure 3 illustrates the Kaplan–Meier survival curves with log-rank tests for the cumulative hazard plot of specific disease-free status between the comparison and migraine group. The results of the log-rank test indicated that migraine patients aged over 55 years could develop dementia more frequently than patients who were not diagnosed with migraine.

## 4. Discussion

This study was used the nationwide population-based dataset which included the entire medical service utilization history of more than 1 million South Koreans. From KNHIS–NSC, we identify mid- and late-life migraines and their potential to cause all-cause dementia events, such as Alzheimer’s disease or vascular dementia. Thus, in this nationwide, retrospective cohort study, we found that the cumulative incidence of all-cause dementia and Alzheimer’s disease was higher in mid- and late-life patients with migraine than in those without migraine; however, there was no significant link between migraine and vascular dementia. Additionally, we detected a more significant association between diagnoses of mid- and late-life migraine and dementia in men, as well as in individuals with a history of comorbidities and an older age. These findings have important implications for clinicians and for the support provided to patients who are diagnosed with migraine regarding preventative measures for dementia.

Currently, it is well known that obesity, diabetes mellitus, hypertension, and coronary heart disease are important risk factors for dementia [11,12,13,14], while the appropriate treatment of those could reduce this risk [15,16,17]. Therefore, in this study, we adjusted for hypertension, diabetes mellitus, stroke, chronic kidney disease, disorders of lipoprotein metabolism, and other lipidemias as comorbidities. Additionally, the exact pathophysiological mechanism between migraine and dementia is still unclear, although several studies have described that vascular problems and brain structural changes, such as white matter abnormalities, are found in migraine [5,18,19,20,21]. These are thought to be important pathologic findings that contribute to the development of dementia. Thus, we excluded individuals who underwent brain or heart surgeries given that these procedures could influence the development of dementia.

Some studies described the hypoperfusion-induced oxidative stress in endothelial and nerve cells were was induced by vascular problems [22,23]. Other studies also showed that cardiovascular disease could contribute to the brain white matter abnormalities [24,25]. Based on previous studies [22,23,24,25], the hypothesis that migraine is associated with an increased risk of dementia can be reasonably speculated. However, evidence from longitudinal studies associating migraine with dementia show controversial findings [5,6,7,8,9,10]. Our findings are quite comparable with those of several previous studies [6,7,8]. However, contrary to other previous studies, we did not include only older patients—we included patients with migraine who were aged over 55 years. Similar to our study, a Danish cohort study included patients with migraine with a primary diagnosis before the age of 59 years [26]. This study demonstrated a higher rate of dementia events in patients with migraine than in those without migraine [26]. Moreover, it is generally accepted that the increased cardiovascular risk of migraine is a composite effect of multiple parameters, such as hypertension, diabetes mellitus, obesity, and dyslipidaemia [27,28,29,30]. Thus, our results support the hypothesis that migraine in midlife may be a risk factor for dementia in later life, specifically for Alzheimer’s disease.

Interestingly, despite the vascular problems in migraine pathophysiology, we found that migraine was not significantly associated with an increased risk of vascular dementia. Consistent with our results, some prior studies showed no association between migraine and increased vascular dementia [6,8] We believe that this finding may imply the lack of a direct causal link between those because our study matched stroke events in both groups, given that stroke is a major risk factor for vascular dementia [31,32]. In addition, we found a higher adjusted HR for dementia in patients with migraine, specifically in men as well as in individuals with comorbidities and an older age, which are related with vascular risks. Thus, we thought the vascular problems may be an intervening variable between migraine and vascular dementia. However, our findings have some inherent limitations. First, vascular dementia as a single entity (without concomitant Alzheimer’s disease) is much less common than Alzheimer’s disease. Second, Alzheimer’s disease most commonly harbors concomitant vascular dementia pathology, but is classified as Alzheimer’s disease alone; hence, the contribution of migraine to the development of Alzheimer’s disease could be mediated by the underlying vascular component of Alzheimer’s disease.

This study had some unique strengths. First, our incidences and HR of dementia was effectively obtained using a large, national, population-based database. Second, our cohort data included a long observation period compared with other studies. Third, the prior validation study revealed that the KNHIS-NSC data showed a similar prevalence of 20 major diseases for each of the years assessed. Thus, it means that the we could assume the reliability of the KNHIS-NSC data as “fair to good [33,34]”.

Our study also had some notable limitations. First, we could not obtain any specific personal medical data, including body mass index, pathology findings, laboratory data, or behavioral risk factors (the history of smoking or alcohol consumption). Second, the diagnosis of migraine and dementia was based on the ICD-10 diagnostic code, not medical records that include details such as the patients’ medical history and the results of neurocognitive questionnaires. It means that this study has the misclassification bias. To overcome this problem, we only included migraine or dementia patients who were diagnosed by neurologists. Third, we could not guarantee that the age at the first hospital visit for migraine accurately corresponded with the age of migraine onset. Fourth, we could not access specific data such as the duration, frequency, and severity of migraine due to lack of data in our registry; therefore, we were unable to investigate whether the duration, frequency, and severity of migraine may have a differential effect on dementia risk. Fifth, we were unable to adjust for the effect of anti-migraine medication doses, such as ergotamine and triptans. Finally, family history, genetic conditions, and radiographic findings based on magnetic resonance imaging (such as brain structural abnormalities) could affect the potential development of Alzheimer’s disease and vascular dementia. However, to overcome these limitations, we enrolled only patients who were aged 55 years or older. Moreover, we matched migraine and non-migraine groups using propensity scores. However, for these limitations, future clinical studies to provide strong evidence for the link between migraine and dementia would need.

## 5. Conclusions

The present study investigated a possible association between mid- and late-life patients with migraine and the development of dementia. We found that mid- and late-life patients with migraine had a higher risk of developing all-cause dementia, with the risk being greater in men as well as in older patients and those with comorbidities. Additionally, mid- and late-life patients with migraine had a higher risk of Alzheimer’s disease, but not vascular dementia. However, to elucidate the underlying pathophysiological mechanisms, further studies included a wider range of factors and diagnostic criteria are required.

## Figures and Tables

**Table 1:** Characteristics of the study subjects.

Variables	Comparison (*n* = 5888)	Migraine (*n* = 1472)	*p* Value
**Sex**			1.000
Male	1412 (24.0%)	353 (24.0%)	
Female	4476 (76.0%)	1119 (76.0%)	
**Ages (years)**			1.000
55–64	3100 (52.6%)	775 (52.6%)	
65–74	2016 (34.2%)	504 (34.2%)	
≥75	772 (13.1%)	193 (13.1%)	
**Residence**			1.000
Seoul	972 (16.5%)	243 (16.5%)	
Second area	1248 (21.2%)	312 (21.2%)	
Third area	3668 (62.3%)	917 (62.3%)	
**Household income**			1.000
Low (0–30%)	1428 (24.3%)	357 (24.3%)	
Middle (30–70%)	1968 (33.4%)	492 (33.4%)	
High (70–100%)	2492 (42.3%)	623 (42.3%)	
**Comorbidities**			1.000
No	928 (15.8%)	232 (15.8%)	
Yes	4960 (84.2%)	1240 (84.2%)	

Comparison, subjects without migraine; Seoul, the largest metropolitan area; second area, other metropolitan cities; third area, other areas.

**Table 2:** Incidence per 1000 person-years and HR (95% CIs) of dementia between comparison (non-migraine) and migraine group.

Variables	N	Case	Incidence	Unadjusted HR (95% CI)	Adjusted HR (95% CI)	*p* Value
**Group**						
Comparison	5888	686	11.1	1.00 (ref)	1.00 (ref)	
Migraine	1472	191	13.5	1.36 (1.15–1.60) ***	1.37 (1.16–1.61) ***	<0.001
**Sex**						
Male	1765	144	8.4	1.00 (ref)	1.00 (ref)	
Female	5595	733	12.5	1.45 (1.21–1.73) ***	1.44 (1.20–1.72) ***	<0.001
**Ages (years)**						
55–64	3875	215	5.0	1.00 (ref)	1.00 (ref)	
65–74	2520	427	16.9	3.65 (3.10–4.30) ***	3.59 (3.04–4.24) ***	<0.001
≥75	965	235	31.8	8.21 (6.81–9.89) ***	8.19 (6.78–9.89) ***	<0.001
**Residence**						
Seoul	1215	130	10.0	1.00 (ref)	1.00 (ref)	
Second area	1560	168	10.4	1.05 (0.83–1.32)	1.25 (0.99–1.57)	0.059
Third area	4585	579	12.4	1.25 (1.04–1.52) *	1.20 (0.99–1.46)	0.057
**Household income**						
Low (0–30%)	1785	220	12.0	1.00 (ref)	1.00 (ref)	
Middle (30–70%)	2460	279	10.9	0.90 (0.75–1.07)	1.03 (0.86–1.23)	0.762
High (70–100%)	3115	378	11.8	0.98 (0.83–1.16)	0.97 (0.82–1.15)	0.735
**Comorbidities**						
No	1160	81	7.3	1.00 (ref)	1.00 (ref)	
Yes	6200	796	12.3	1.64 (1.31–2.06) ***	1.41 (1.12–1.77) **	0.003

Seoul, the largest metropolitan area; second area, other metropolitan cities; third area, other areas. HR, hazard ratio; CI, confidence interval. * *p* < 0.05, ** *p* < 0.010, and *** *p* < 0.001.

**Table 3:** Hazard ratios of dementia by sex among the sample patient.

Sex	Male		Female	
	Comparison	Migraine	Comparison	Migraine
**Dementia**				
Unadjusted HR (95% CI)	1.00 (ref)	1.98 (1.37–2.86) ***	1.00 (ref)	1.24 (1.04–1.49) *
Adjusted HR (95% CI)	1.00 (ref)	2.07 (1.44–2.99) ***	1.00 (ref)	1.25 (1.04–1.49) *

HR, hazard ratio; CI, confidence interval. * *p* < 0.05, and *** *p* < 0.001.

**Table 4:** Hazard ratios of dementia by comorbidities among the sample patient.

Comorbidities	No		Yes	
	Comparison	Migraine	Comparison	Migraine
Dementia				
Unadjusted HR (95% CI)	1.00 (ref)	1.19 (0.69–2.04)	1.00 (ref)	1.38 (1.16–1.63) ***
Adjusted HR (95% CI)	1.00 (ref)	1.21 (0.70–2.07)	1.00 (ref)	1.38 (1.17–1.64) ***

HR, hazard ratio; CI, confidence interval. *** *p* < 0.001.

**Table 5:** Hazard ratios of dementia by age among the sample patient.

Ages	55–64		65–74		≥75	
	Comparison	Migraine	Comparison	Migraine	Comparison	Migraine
**Dementia**						
Unadjusted HR (95% CI)	1.00 (ref)	1.11 (0.78–1.58)	1.00 (ref)	1.43 (1.14–1.80) **	1.00 (ref)	1.52 (1.12–2.07) **
Adjusted HR (95% CI)	1.00 (ref)	1.11 (0.78–1.58)	1.00 (ref)	1.43 (1.14–1.80) **	1.00 (ref)	1.50 (1.10–2.03) **

HR, hazard ratio; CI, confidence interval. ** *p* < 0.010.

**Table 6:** Incidence per 1000 person-years and HR (95% CI) of specific diseases (Alzheimer’s disease and vascular dementia).

Variables	N	Case	Incidence	Unadjusted HR (95% CI)	Adjusted HR (95% CI)
**Alzheimer’s disease**					
Comparison	5888	521	8.4	1.00 (ref)	1.00 (ref)
Migraine	1472	137	9.6	1.31 (1.08–1.58) **	1.31 (1.08–1.58) **
**Vascular dementia**					
Comparison	5888	124	2.0	1.00 (ref)	1.00 (ref)
Migraine	1472	38	2.6	1.41 (0.98–2.03)	1.42 (0.98–2.05)

HR, hazard ratio; CI, confidence interval. ** *p* < 0.010.

## Data Availability

The authors confirm that the data supporting the findings of this study are available within the article.

## Supplementary Materials

The following are available online at https://www.mdpi.com/article/10.3390/jpm11100990/s1, Table S1: Description of time to event and censored data
