# Supplementary material for: Mid- and Late-Life Migraine Is Associated with an Increased Risk of All-Cause Dementia and Alzheimer’s Disease, but Not Vascular Dementia: A Nationwide Retrospective Cohort Study
Source: J Pers Med. 2021 Sep 30;11(10):990. doi: 10.3390/jpm11100990 (PMC8540823; doi:10.3390/jpm11100990)
Supplement: Supplementary file 1 [file jpm-11-00990-s001.zip › jpm-1392091-supplementary.pdf]

**Table S1.** Description of time to event and censored data.

| | The Number of Dementia Event |
|---|---|
| **Event** | 877 |
| Comparison | 686 |
| Migraine | 191 |
| **Total censored (No event)** | 6483 |
| Comparison | 5202 |
| Migraine | 1281 |
| **Termination of study** | 5243 |
| Comparison | 4156 |
| Migraine | 1087 |
| **Loss to follow up/Drop-out** | 1240 |
| Comparison | 1046 |
| Migraine | 194 |
